# Aging impairs peroxisome biogenesis in human B cells

**DOI:** 10.1093/gerona/glaf148

**Published:** 2025-07-09

**Authors:** Jacinta Correia, Promit Sinha Roy, Kaitlyn G Holden, Marian L Kohut, Hua Bai

**Affiliations:** Department of Genetics, Development, and Cell Biology, Iowa State University, Ames, Iowa, United States; Molecular, Cellular and Developmental Biology Program, Iowa State University, Ames, Iowa, United States; Department of Kinesiology, Iowa State University, Ames, Iowa, United States; Nanovaccine Institute, Iowa State University, Ames, Iowa, United States; Department of Kinesiology, Iowa State University, Ames, Iowa, United States; Nanovaccine Institute, Iowa State University, Ames, Iowa, United States; Department of Genetics, Development, and Cell Biology, Iowa State University, Ames, Iowa, United States

**Keywords:** Peroxisome biogenesis, Peroxisomal enzyme import, B cell aging, B cell metabolism

## Abstract

Emerging evidence highlights the critical role of cellular metabolism in immune cell activation, development, and function. Peroxisomes, key metabolic organelles, maintain metabolic homeostasis, yet their role in immune cells remains underexplored. While animal studies show age-related declines in peroxisome biogenesis, this process is unconfirmed in human aging. We investigated peroxisome biogenesis in human peripheral blood mononuclear cells (PBMCs) and found a significant decline in aged CD19^+^ B cells compared to CD4^+^ T cells, CD8^+^ T cells, and CD14^+^ monocytes. B cell aging also reduces peroxisomal matrix enzyme import, evidenced by decreased SKL-containing enzymes and mature ACOX1, alongside downregulation of PEX19 and E3 ubiquitin ligases PEX2, PEX10, and PEX12. These findings confirm an evolutionarily conserved and age-related decline in peroxisome biogenesis. Further, our work unveils cell type-specific changes in aging human PBMCs, and provides new insights into peroxisome-mediated immunometabolism and B cell aging.

## Introduction

Aging is the primary risk factor for most diseases that limit health span.^1^ With increased age, individuals undergo numerous cellular and molecular changes such as chronic inflammation, cellular senescence, stem cell exhaustion, mitochondrial dysfunction, loss of proteostasis, epigenetic alterations, and genomic instability.^1^ Among these hallmarks of aging, loss of organelles homeostasis has emerged as a vital cause of tissue aging.^2^ For instance, mitochondrial dynamics is an important adaptive stress response responsible for repairing and recycling damaged mitochondria and restoring cellular homeostasis.^3^ Recently, mitochondrial dynamics has emerged as a novel regulator of aging.^4–6^ Mitochondria dynamics often alters during normal aging,^4^ while muscle-specific overexpression of dynamin-related protein Drp1 prolongs *Drosophila* lifespan and healthspan.^5^ However, the age-related alterations of many subcellular organelles (such as peroxisomes) and their impacts on aging and age-related diseases are largely unexplored.

Recent studies from our groups and others revealed that peroxisomal function is dysregulated during aging, which contributes to age-related chronic inflammation and various age-related pathologies.^6–10^ Peroxisomes are single membrane-bound organelles that play crucial roles in regulating cellular redox homeostasis, oxidation of very long chain fatty acids (VLCFAs), and biosynthesis of ether phospholipid (e.g., plasmalogen).^11^^,^^12^ Because peroxisomal matrix proteins are synthesized in the cytosol, all peroxisomal functions are dependent on the import of matrix proteins into the organelle, which is controlled by a set of peroxisomal biogenesis proteins named peroxins (PEXs).^13–17^ Genetic mutations in PEX genes often disrupt peroxisomal biogenesis and protein import, which can lead to severe impairment of tissue functions as seen in human peroxisome biogenesis disorder (PBD).^18^^,^^19^ About 30 conserved peroxins are known to regulate peroxisome biogenesis, including pre-peroxisomal vesicle formation, import of peroxisomal membrane proteins and matrix enzymes, and peroxisomal growth and division.^12^^,^^18^^,^^20^ Peroxisomal membrane protein (PMP) receptor PEX19, together with PEX3 and PEX16, recruits PMPs containing membrane peroxisome targeting signals (PTS) to the peroxisomal membrane.^21^ PEX19 is also involved in *de novo* biogenesis of pre-peroxisomal vesicle.^13^^,^^21^

Peroxisome biogenesis involves a specialized import machinery that translocates folded and oligomerized matrix proteins into peroxisomes. This machinery includes receptor proteins PEX5 and PEX7, which recognize peroxisomal targeting signals (PTS1 and PTS2) on matrix proteins, a docking complex (PEX13, PEX14), and a receptor recycling system (e.g., E3 ubiquitin ligase complex, PEX2, PEX10, PEX12).^13^^,^^16^^,^^17^^,^^22–24^Although age-related decline of peroxisome biogenesis has been reported in animal models and cultured senescent human fibroblasts,^7–9^^,^^25^ it remains to be determined whether this dysregulation occurs during human aging.

While peroxisomes are critical for fatty acid β-oxidation and have been implicated in B cell development and antibody production in mice,^26^^,^^27^ their role in B cell metabolism and human immunosenescence remains underexplored. Immunosenescence, the age-dependent decline of the immune system, is marked by reduced immune responses to infections and vaccinations, alongside increased autoimmune reactivity.^28^^,^^29^ In aged B cells, antibody production efficiency decreases, and B cell differentiation is impaired due to reduced expression of key transcription factors (E2A, PAX5) and pre-B cell receptor.^30–33^ Additionally, age-associated B cells (ABCs) or double-negative memory B cells produce proinflammatory cytokines and autoantibodies, exhibiting a metabolic shift toward oxidative phosphorylation and aerobic glycolysis.^34–36^ Emerging evidence has revealed a vital role of cellular metabolism in immune cell development and function.^37^ Despite their important role in fatty acid beta-oxidation, peroxisomes are relatively unexplored in B cell metabolism. A recent study suggests that peroxisomes are crucial for the development of marginal zone and B1 B cells and antibody production in mice, without altering T cell development.^38^

To gain insights into the role of peroxisomes in B cell aging, we examined how aging affects peroxisome biogenesis and protein import in human peripheral blood mononuclear cells (PBMCs), with a focus on B cells. We find that peroxisomes are equally present in all cell types tested. Interestingly, old CD19^+^ B cells, but not CD4^+^ T cells, CD8^+^ T cells, and CD14^+^ monocytes, exhibited significant decline in both peroxisome biogenesis and peroxisomal protein import. Our findings uncover age-related and cell type-dependent changes in peroxisome biogenesis and import function, which can shed new insights into the understanding of peroxisome-mediated immunometabolism and B cell aging.

## Methods

### Human participants

A total of 14 individuals were recruited to participate in this study. Participants were classified into two distinct age groups: young adult (19- to 35-year old, *n* = 7, four females and three males) and older adult (60-to 74-year old, *n* = 7, three females and four males; Table S1). At the initial study visit, a health questionnaire was completed to determine eligibility for participation. Individuals with an immune disorder, cancer, or treatment with any medication known to alter immune response were excluded. If participants were unsure whether a condition or medication might be an exclusion criterion, a list of common conditions or medications was provided for the participant to examine, and clarification by the researcher was provided as needed. Categories of medications that would be expected to interfere substantially with an immune response or immunometabolism include glucocorticoids, mTOR inhibitors, calcineurin inhibitors, antimetabolites, cytokine modulators, TNFα antagonists, and nucleotide synthase inhibitors. This list provided examples of medications and categories that warrant exclusion but was not intended to be an all-encompassing list. Metformin warranted exclusion, not as an immunosuppressive medication, but one that could significantly alter immunometabolic responses. Due to their widespread use and the mixed or modest findings in response to immunomodulatory properties, Statins did not warrant exclusion. Similarly, over-the-counter low-dose aspirin therapy and NSAID usage warranted exclusion. All procedures involving human subjects were approved by the Institutional Review Board at Iowa State University.

### Isolation of PBMCs and CD19^+^ B cells

PBMCs were isolated via centrifugation of PBS-diluted whole blood (1:1 PBS: blood) over a Ficoll^®^Paque Plus density gradient (Sigma-Aldrich Inc.) per manufacturer’s instructions. Next, B cells were isolated by incubating PBMCs with CD19 magnetic microbeads (Miltenyi Biotec), followed by subsequent magnetic separation of the CD19^+^ cells over a MACS^®^ Column. The flowthrough PBMCs containing T cells and monocytes were also collected.

### Immunofluorescence staining

Cells were first fixed with 4% paraformaldehyde (Electron Microscopy Sciences) in phosphate buffered saline (PBS) overnight at 4 °C. After three washes with 1x PBS with 0.3% Triton X-100 (0.3% PBST), the cells were blocked with 5% normal donkey serum (Jackson ImmunoResearch) for 1 h. The cells were stained with primary antibodies diluted in 5% NDS for 16-24 h at 4 °C. On the next day, cells were washed with 0.3% PBST and incubated with secondary antibodies and Hoechst 33342 for 1 h at room temperature. The cells were then washed and mounted on the slides using ProLong Diamond antifade reagent (Thermo Fisher Scientific). The samples were imaged with an FV3000 Confocal Laser Scanning Microscope (Olympus). The primary and secondary antibodies are summarized below: anti-CD19 (Proteintech #66298-1-Ig, 1:200), anti-CD14 (Proteintech #60253-1-Ig, 1:200), anti-CD4 (Proteintech #67786-1-Ig, 1:200), anti-CD8 (Proteintech #66868-1-Ig, 1:200), anti-SKL (Gift from Richard Rachubinski lab, 1:200), CoraLite^®^594-conjugated PEX14 Polyclonal antibody (Proteintech # CL594-10594, 1:200), Alexa Flour^®^ 488 anti-mouse IgG (Jackson ImmunoResearch #715-545-150, 1:500), Alexa Fluor^®^ 594 anti-Rabbit IgG (Jackson ImmunoResearch #711-585-152, 1:500).

### Western blotting

The proteins were extracted using NP-40 cell lysis buffer (Thermo Fisher Scientific) containing 1X protease inhibitor cocktail (Sigma). Protein samples were denatured with Laemmli sample buffer (Bio-Rad) at 95 °C for 5 min. Then, the protein samples were separated by Mini-PROTEAN^®^ TGX Precast Gels (Bio-Rad Laboratories), transferred to PVDF membranes, immunoblotted with the following primary and secondary antibodies, and visualized with Pierce ECL Western Blotting Substrate. (Thermo Fisher Scientific). See below for the list of primary and secondary antibodies: anti-PEX14 (Proteintech #10594-1-AP, 1:2000), anti-PEX19 (Proteintech #14713-1-AP, 1:2000), anti-PEX2 (Proteintech #22163-1-AP, 1:2000), anti-PEX10 (Proteintech #27502-1-AP, 1:2000), anti-PEX12 (Proteintech #27011-1-AP, 1:2000), anti-ACOX1 (Proteintech #10957-1-AP, 1:2000), anti-β-Actin (Cell signaling technology #4967, 1:2000), Peroxidase anti-Rabbit IgG (Jackson ImmunoResearch #711-035-152, 1:5000).

### Image analysis and quantification

Confocal images were processed and analyzed using Fiji ImageJ and Olympus cellSens Dimensions software. A total of 10 images were quantified per sample. The number of PEX14 punctae were quantified using the count & measure function in Olympus cellSens Dimensions software. The colocalization between PEX14 and SKL was measured by using the coloc2 plug-in of ImageJ and presented as Pearson correlation coefficient.

### Statistical analysis

GraphPad Prism (GraphPad Software v7.05) was used for statistical analysis. Unpaired two-tailed Student’s *t* test or one-way ANOVA (Holm–Sidak comparison) was performed to compare the mean value between young and old groups. Linear regression analysis was performed to examine the linear relationship between age and peroxisome function. *R* square values were shown in each linear regression graph. Data are represented as mean ± SD in each figure.

## Results

### Peroxisome biogenesis is impaired in aged B cells

Age-related decline of peroxisome biogenesis has been previously observed in animal models and cultured senescent human fibroblasts,^7–9^^,^^25^ however, to date, there is no attempt to directly measure peroxisome biogenesis in any human tissues during aging. To address this knowledge gap, we thoughts to monitor peroxisome biogenesis in PBMCs isolated from young (19- to 35-year old) and old individuals (60- to 74-year old; Table S1), because PBMCs are one of easily accessible sources of human tissues and cells that exhibit significant cellular and metabolic changes during aging.

**Figure 1.**
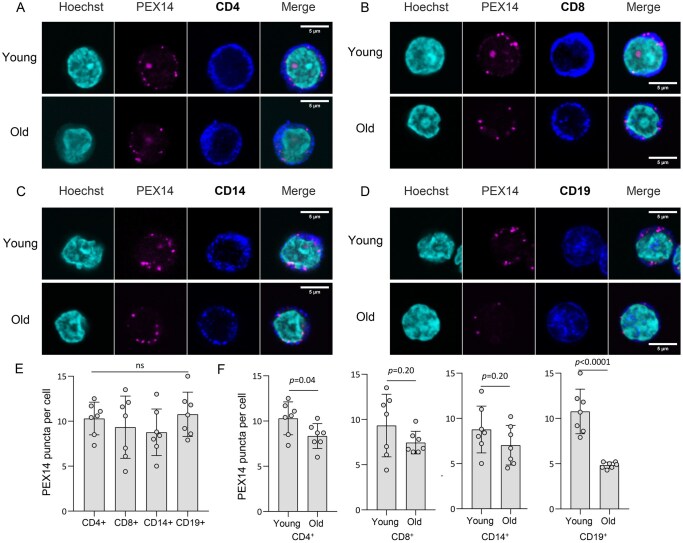
Peroxisome number decreases in human immune cell aging. **(**A) Immunofluorescence staining of PEX14 in CD4^+^ T cells isolated from young (23-year old) and old individuals (74-year old). Hoechst 33342 was used for nuclear staining. Scale bar: 2 μm. (B) Immunofluorescence staining of PEX14 in CD8^+^ T cells isolated from young (23-year old) and old individuals (74-year old). Scale bar: 2 μm. (C) Immunofluorescence staining of PEX14 in CD14^+^ T cells isolated from young (23-year old) and old individuals (74-year old). Scale bar: 2 μm. (D) Immunofluorescence staining of PEX14 in CD19^+^ T cells isolated from young (23-year old) and old individuals (74-year old). Scale bar: 2 μm. (E) Quantification of PEX14-positive puncta in young individuals. one-way ANOVA, ns: not significant, *n* = 7. (F) Quantification of PEX14-positive puncta in four immune cell types isolated from young and old individuals. Student’s *t*-test, *n* = 7. Data are represented as mean ± SD. Each data point (circle in the graphs) represents an individual participant, categorized into two age groups: young (19- to 35-year old) and old (60- to 74-year old).

We first measured the peroxisome abundance in different immune cell types through immunofluorescence staining with the antibody against PEX14, the central membrane-bound component of the peroxisome docking complex and a common peroxisome marker.^24^^,^^39^ To distinguish different immune cell types from isolated PBMCs, we co-stained PEX14 with CD4 (helper T cells), CD8 (cytotoxic T cells), CD19 (B cells), or CD14 (monocytes), respectively. We found that peroxisomes are present in all immune cells (Figure 1A-D). The number of peroxisomes indicated by the number of PEX14 puncta, is similar among all immune cells tested, about 10 PEX14 puncta per cell in young individuals (Figure 1E). It indicates that peroxisomes are abundant in PBMCs. Next, we asked whether aging alters the peroxisome biogenesis in PBMCs. Interestingly, the number of PEX14 puncta in aged CD19^+^ B cells was two-fold less than that in young CD19^+^ B cells (Figure 1F), while the reduction of PEX14 puncta in aged CD4^+^ T cells was moderate **(**Figure 1F). There were no significant changes of PEX14 puncta in CD8^+^ T cells and CD14^+^ monocytes during aging (Figure 1F). These data demonstrated a cell type- and age-dependent decline in peroxisome biogenesis during immune cell aging, while CD19^+^ B cells exhibit a strong impairment in peroxisome biogenesis.

**Figure 2.**
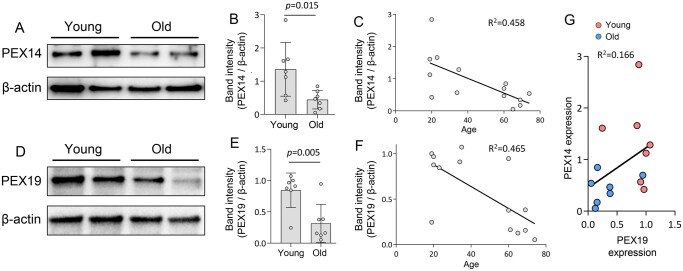
Peroxisome biogenesis factor PEX19 is downregulated in aged B cells. **(**A) Western blot analysis of PEX14 in young and old CD19^+^ B cells. (B) The quantification of band intensity of PEX14 normalized to beta-actin. Student’s *t*-test, *n* = 7. Data are represented as mean ± SD. (C) Linear regression plot showing the correlation relationship between PEX14 expression and age. (D) Western blot analysis of PEX19 in young and old CD19^+^ B cells. (E) The quantification of band intensity of PEX19 normalized to beta-actin. Student’s *t*-test, *n* = 7. Data are represented as mean ± SD. (F) Linear regression plot showing the correlation relationship between PEX19 expression and age. (G) Linear regression plot showing the correlation relationship between PEX14 and PEX19 protein expression. Each data point (circle in the graphs) represents an individual participant, categorized into two age groups: young (19- to 35-year old) and old (60- to 74-year old).

We further verified our immunostaining results using western blotting and CD19^+^ B cell-enriched PBMCs. As shown in Figure 2A and B (Figure S1), the expression levels of PEX14 protein were significantly downregulated (more than 2-fold) in CD19^+^ B cells isolated from old individuals. Linear regression plot showed a strong correlation between age and PEX14 protein expression (*R*^2^ = 0.458; Figure 2C**)**. Thus, like what observed in animal models, peroxisome biogenesis also declines with human aging, especially in aged B cells.

### Peroxisome biogenesis factor PEX19 is downregulated in aged B cells

The changes in peroxisome number could be due to the reduction of peroxisome biogenesis or the induction of pexophagy. To further confirm the reduction of peroxisome biogenesis process in aged B cells, we measured the protein expression of PEX19, the central peroxisome biogenesis factors.^21^ PEX19 is known to be required for pre-peroxisomal vesicle formation and PMP trafficking to peroxisomal membrane.^13^^,^^21^ We performed western blotting using the proteins extracted from column enriched CD19^+^ B cells and found that PEX19 protein expression was significantly decreased in aged B cells (Figure 2D-E and Figure S2). Further, linear regression analysis suggests a strong correlation between age and PEX19 protein expression (*R*^2^ = 0.465; Figure 2F). The age-dependent changes of PEX19 expression positively correlates with PEX14 expression in both young and old groups (Figure 2G). Together, our data demonstrates that peroxisome biogenesis is dysregulated during B cell aging.

### The import of peroxisomal matrix enzymes declines during B cell aging

**Figure 3.**
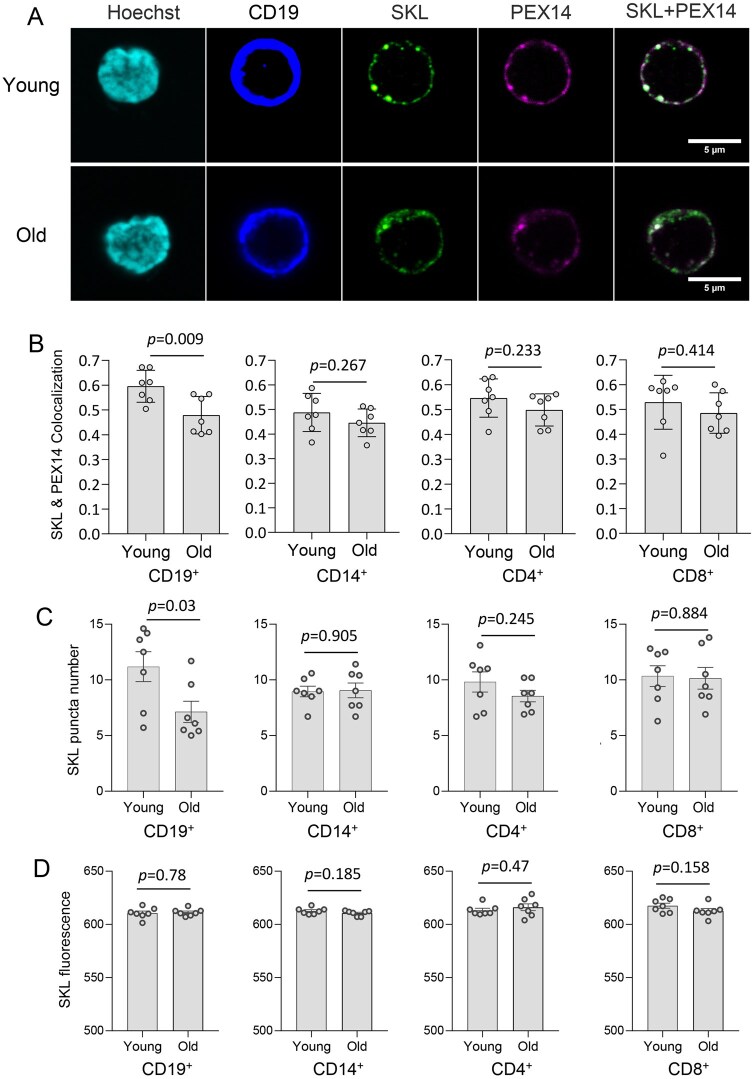
The import of peroxisomal matrix enzymes declines during B cell aging. (A) Immunofluorescence staining of SKL and PEX14 in CD19^+^ B cells isolated from young (23-year old) and old individuals (74-year old). Hoechst 33342 was used for nuclear staining. Scale bar: 2 μm. (B) Pearson correlation coefficient (*r*) to quantify SKL and PEX14 co-localization in four immune cell types with age. (C) Quantification of SKL puncta number in four immune cell types with age. (D) Quantification of SKL fluorescence intensity in four immune cell types with age. Student’s *t*-test, *n* = 7. Data are represented as mean ± SD. Each data point (circle in the graphs) represents an individual participant, categorized into two age groups: young (19- to 35-year old) and old (60- to 74-year old).

Besides pre-peroxisomal vesicle formation and peroxisomal membrane protein trafficking, peroxisome biogenesis, the import (or translocation) of peroxisomal matrix enzymes from the cytosol into peroxisomes is critical for the biogenesis of mature and functional peroxisomes.^12^^,^^13^ We previously show that the import of peroxisomal matrix enzymes declines in aged hepatocytes in *Drosophila.*^9^ To monitor peroxisomal enzyme import in aged PBMCs, we performed immunostaining using an anti-SKL antibody that recognizes the SKL tripeptide (PTS1 signal peptide) of the majority of peroxisomal matrix enzymes. We co-stained SKL with PEX14 to locate peroxisomes. The co-localization between SKL and PEX14 was significantly reduced in aged CD19^+^ B cells, but not in other immune cell types (Figure 3A-B). In addition, the number of SKL positive punctae was significantly reduced in aged PBMCs, especially in CD19^+^ B cells (Figure 3A and C), while the SKL intensity remained no change (Figure 3D). Thus, less SKL signal (mark for peroxisomal enzyme import) was detected in PEX14-positive peroxisomes during B cell aging. These findings suggest that import of peroxisomal matrix enzymes declines during B cell aging.

**Figure 4.**
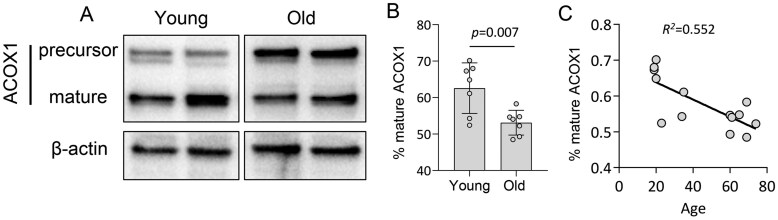
The import of ACOX1 declines during B cell aging. (A) Western blot analysis of ACOX1 translocation in young and old CD19^+^ B cells. (B) The quantification of band intensity of ACOX1 (mature form normalized to total ACOX1). Student’s *t*-test, *n* = 7. Data are represented as mean ± SD. (C) Linear regression plot showing the correlation relationship between mature ACOX1 and age. Each data point (circle in the graphs) represents an individual participant, categorized into two age groups: young (19- to 35-year old) and old (60- to 74-year old).

To further validate the above findings, we performed western blotting to directly test the translation of peroxisomal matrix enzyme ACOX1 (Acyl-CoA Oxidase 1), the key enzyme involved in VLCFA beta-oxidation.^40^ The maturation of ACOX1 is regulated through proteolytical processing in peroxisomes, which converts a precursor protein (75 kDa) into a mature form (52 kDa).^41^ Thus, the ratio between mature ACOX1 and precursor (or the proportion of mature protein) can be used as an indicator of peroxisomal enzyme import efficiency. We found that the proportion of mature ACOX1 was significantly decreased in aged B cells (Figure 4A and B and Figure S3), which suggests that the translocation of ACOX1 protein is reduced during B cell aging. In addition, linear regression analysis showed a strong correlation between age and ACOX1 maturation (*R*^2^ = 0.552; Figure 4C), which further supports an age-dependent decline of peroxisomal enzyme import in human B cells.

### Peroxisomal E3 ubiquitin ligases are downregulated in aged B cells

**Figure 5.**
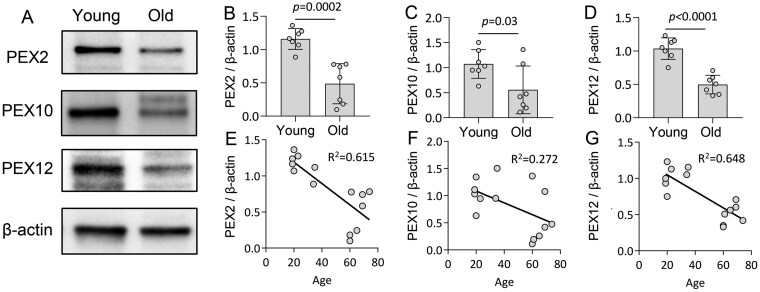
Peroxisomal E3 ubiquitin ligases are downregulated in aged B cells. (A) Western blot analysis of PEX2, PEX10, and PEX12 in young and old CD19^+^ B cells. (B-D) The quantification of band intensity of PEX2, PEX10, and PEX12 normalized to beta-actin. Student’s *t*-test, *n* = 7. Data are represented as mean ± SD. (E-G) Linear regression plot showing the correlation relationship between mature E3 ubiquitin ligases and age. Each data point (circle in the graphs) represents an individual participant, categorized into two age groups: young (19- to 35-year old) and old (60- to 74-year old).

Age-related decreases in peroxin expression might explain the decline of peroxisomal enzyme import in aged human B cells, for example, the downregulation of key peroxisome translocon component PEX14 observed in the present study might contribute to the reduction of peroxisomal enzyme import. Conversely, an early study reported that PEX5 recycling is blocked in senescent human fibroblast culture, which leads to an accumulation of PEX5 protein on peroxisomal membrane and a reduction of peroxisomal enzyme import.^25^ After each peroxisomal enzyme import, PEX5 typically recycles back to cytosol through monoubiquitination of the protein. The monoubiquitination of PEX5 is controlled by a membrane-embedded, RING-type E3 ubiquitin ligase complex composed of PEX2, PEX10, and PEX12.^13^ We hypothesized that the downregulation of the E3 ubiquitin ligases might contribute to the decline of peroxisomal enzyme import function. To test this idea, we measured the protein expression of PEX2, PEX10, and PEX12 using western blotting and column enriched CD19^+^ B cells. As shown in Figure 5A-D (Figure S4), the expression of all three E3 ubiquitin ligases were significantly reduced in aged B cells. Further, linear regression analysis showed a strong correlation between age and the expression of PEX2 and PEX12 (*R*^2^ = 0.615 and 0.648), but a weak correlation between aging and PEX10 expression (*R*^2^ = 0.272; Figure 5E-G). Thus, our data suggests that an age-dependent decrease in E3 ubiquitin ligase complex might be one of the critical causes of dysregulated peroxisomal enzyme import during human B cell aging.

## Discussion

Although impaired peroxisome biogenesis has been reported in animal models and cultured human fibroblasts,^7–9^^,^^25^ it has not been assessed in aged humans. The present study characterized the impact of aging on peroxisome biogenesis in human PBMCs, the easily accessible cells. Through cellular and molecular characterization, we show that peroxisome biogenesis declined significantly in aged CD19^+^ B cells, when compared to CD8^+^ T cells and CD14^+^ monocytes. The decline of peroxisome biogenesis in CD4^+^ T cells was moderate but significant. The decline of peroxisome biogenesis in aged CD19^+^ B cells was accompanied by a significant decrease in biogenesis factor PEX19. In addition, we show that the import of peroxisomal matrix enzymes declines during B cell aging, as indicated by decreased co-localization between SKL and PEX14 and reduction of mature ACOX1. One possible contribution of reduced peroxisomal enzyme import could be the downregulation of three E3 ubiquitin ligases, PEX2, PEX10, and PEX12, which might lead to blockage of PEX5 monoubiquitination and recycling. Our findings align well with a recent mouse study,^38^ both of which reveal a vital role of peroxisome biogenesis in B cell development and function. In addition, our data demonstrates that like animal models, aging also impairs peroxisome biogenesis in humans, particularly in aged B cells. Therefore, age-related decline of peroxisome biogenesis is an evolutionarily conserved process. Peroxisome biogenesis can be a novel biomarker of aging, and potentially a target for treating age-related diseases, as well as immunosenescence.

Peroxisomes are generated through multiple interconnected processes, including pre-peroxisomal vesicle formation (*de novo* biogenesis), import of peroxisomal membrane proteins, import of peroxisomal matrix enzymes, and division of pre-existing peroxisomes.^12^^,^^18^^,^^20^ PEX19 is a cytoplasmic chaperon and PMP receptor that controls the initial steps of peroxisome biogenesis.^13^^,^^21^ PEX19 is farnesylated at a C-terminal cysteine residue in yeast, which is essential for its function in peroxisome biogenesis.^42^ The pre-peroxisomal vesicles originate from the ER containing PEX16, which binds to PEX3, a docking receptor for PEX19.^21^^,^^43^ In *Drosophila*, loss of *Pex19* was associated with accumulation of VLCFAs, depletion of shorter fatty acids, mitochondrial abnormalities, neurodegeneration, and reduced viability.^44^ Hyperactive of hepatocyte nuclear factor 4 (Hnf4) signaling is likely the cause of aberrant lipid metabolism observed in *Pex19* mutants.^44^ Increased acid lipase expression and accumulation of free fatty acids were also present in a PEX19-deficient patient skin fibroblast line.^44^ In the present study, we found that PEX19 protein levels were downregulated during B cell aging. The loss of PEX19-mediated peroxisome biogenesis with age might contribute to the dysregulation of B cell development and function by altering B cell metabolism.

During peroxisome maturation process, peroxisomal enzymes are imported from cytosol into peroxisome by first binding to cytosolic receptor PEX5, and then, docking with the membrane translocon complex that consists of PEX14 and PEX13.^13^^,^^23^^,^^24^ The expression of PEX14, the core component of peroxisome translocon and the common peroxisome marker, is often downregulated with age,^7^^,^^8^^,^^45^ although contrary results were reported in mouse hippocampus where PEX14 was induced as an adaptive response to oxidative stress in aging and neurodegenerative disease model.^46^ Mutations in PEX14 have been found in a few patients with Zellweger syndrome and peroxisome biogenesis deficiency.^47^^,^^48^ The downregulation of PEX14 in aged human B cells indicates an impairment of peroxisome biogenesis, as well as a defect in peroxisomal enzyme import.

Recent studies by our group and others have demonstrated that aging impairs peroxisomal enzyme import function in *C. elegans,*^7^  *Drosophila,*^9^ and cultured human fibroblasts.^25^ Most peroxisomal enzymes contain a C-terminal PTS1 signal, also known as the SKL sequence, which is recognized by cytosolic receptors. Similar to the decline of peroxisomal enzyme import observed in animal models, we found that during B cell aging, PEX14-positive peroxisomes significantly lacked SKL-containing proteins, implying a reduction in peroxisomal enzymes import in aged human B cells. In addition, we observed an age-dependent decrease in E3 ubiquitin ligase complex (PEX2, PEX10, and PEX12) during human B cell aging, suggesting an impaired PEX5 monoubiquitination and recycling. Our findings align with the previous studies showing accumulated PEX5 proteins on peroxisome membrane and the blockage of PEX5 recycling in senescent human fibroblasts.^25^ Previous studies show that *Pex2* mutants significantly altered carbohydrate metabolism in *Drosophila*, such as glycolysis, glycogen metabolism, and pentose phosphate pathway.^49^ The connection between peroxisome deficiency and carbohydrate metabolism is unexpected, since carbohydrate metabolism does not involve peroxisomal enzymes. Although similar carbohydrate metabolic alterations were observed in mouse *Pex5* mutants.^50^ It is known that carbohydrate metabolism plays an essential role in B cell development. B cell activation is often associated with increased glucose uptake, which provides fuel for nucleotide and fatty acid synthesis.^51^^,^^52^ Removing glucose from the culture media blocks B cell proliferation and differentiation upon stimulation. The downregulation of peroxisome E3 ubiquitin ligase complex (PEX2-PEX10-PEX12) could alter B cell carbohydrate metabolism, which will lead to impaired B cell development and maturation, and eventually decreased antibody production in old age.

Besides carbohydrate metabolism, age-dependent decline of peroxisome biogenesis could impact B cell function via altering cellular lipid metabolism, including VLCFA beta-oxidation. In the present study, we found the translocation of ACOX1, the key enzyme involved in peroxisomal VLCFA beta-oxidation,^40^ is significantly reduced during B cell aging. As a consequence, loss of peroxisomal import of ACOX1 could lead to accumulation of VLCFA, induction of oxidative stress and lipotoxicity, mitochondrial dysfunction (e.g., impaired mitochondrial beta-oxidation).

Although our knowledge on immune cell dysfunction in human peroxisome biogenesis disorders is limited, a few recent studies show that peroxisome gene knockout in mice alters lymphoid and myeloid functions.^53–55^ Intriguingly, one recent study reports impaired development of marginal zone and B1 B cells, but not T cell development, in *Pex5* knockout mice,^38^ suggesting a differential regulation of lymphoid cell function by peroxisomes. In recent years, emerging evidence suggests a strong link between metabolic homeostasis and B cell function and development.^56^^,^^57^ The metabolic demands differ greatly in different B cell subsets or different lymphoid cells.^58^^,^^59^ It might be possible that high energy demands of B cells rely on strong peroxisomal and mitochondrial fatty acids β-oxidation, while T cells and monocytes may depend on alternative metabolic pathways. The cell-type-specific metabolic demands could explain the differences in age-dependent peroxisome dysregulation among different PBMC cells.

In summary, our study reveals that age-dependent decline of peroxisome biogenesis is conserved in human aging, at least in aging B cells. Our findings uncover an age-related and cell type-dependent change in peroxisome biogenesis and shed new insights into the understanding of peroxisome-mediated immunometabolism and B cell aging. Future studies will investigate the causal link between age-related peroxisome impairment and B cell function using mouse models, while also exploring metabolic differences across lymphoid cell types.

### Limitation of the study

One of the limitations of the study is the small sample size. We recognize that the research involved in human participants requires large sample size (100 individual or above), due to large genetic and environmental variations associated with diverse human populations. However, given that multiple markers of peroxisome biogenesis show robust negative correlation with human age, we are confident about the potential impact of aging on peroxisome biogenesis in human PBMCs, particularly CD19^+^ B cells.

The second limitation of this study is the age-dependent changes in hematopoiesis, which may strongly affect B cell renewal in circulation. As we age, the ability of hematopoietic stem cells (HSCs) in the bone marrow to produce new B and T cells declines,^60^ leading to a shift toward myeloid cells like neutrophils and monocytes.^61^^,^^62^ This reduces the production of naïve lymphocytes, while memory cells, which have previously encountered specific pathogens, expand. Peroxisome biogenesis is likely regulated differently in different subsets of B cells. Additional B cell markers beyond CD19 will be needed to fully characterize the age-dependent changes of peroxisome biogenesis in different B cell subsets.

The third limitation is that the biological age of B cells is influenced by their developmental stage, the number of immune responses they’ve been involved in, and changes in their function over time. In the context of an aging immune system, B cells may show signs of reduced effectiveness, but their “age” is a dynamic concept, influenced by both intrinsic developmental processes and extrinsic factors like exposure to pathogens or vaccines. Thus, future studies are needed to decipher developmental stage-dependent alterations in peroxisome biogenesis during human B cell aging.

## Supplementary Material

glaf148_Supplementary_Data
