# Supplementary material for: Aging impairs peroxisome biogenesis in human B cells
Source: J Gerontol A Biol Sci Med Sci. 2025 Jul 9;81(2):glaf148. doi: 10.1093/gerona/glaf148 (PMC12832945; doi:10.1093/gerona/glaf148)
Supplement: glaf148_Supplementary_Data [file glaf148_supplementary_data.zip › suppl_data/PBMC Project figures_Main_v3b_suppl.pdf]

SUPPLEMENTAL MATERIALS

Supplementary Table 1. Participant information

Age Group	Participant ID	Age	Sex
Young	1	19	Male
Young	2	19	Female
Young	3	20	Female
Young	4	20	Male
Young	5	23	Female
Young	6	34	Male
Young	7	35	Female
Old	8	60	Female
Old	9	60	Male
Old	10	61	Female
Old	11	65	Male
Old	12	69	Male
Old	13	69	Male
Old	14	74	Female

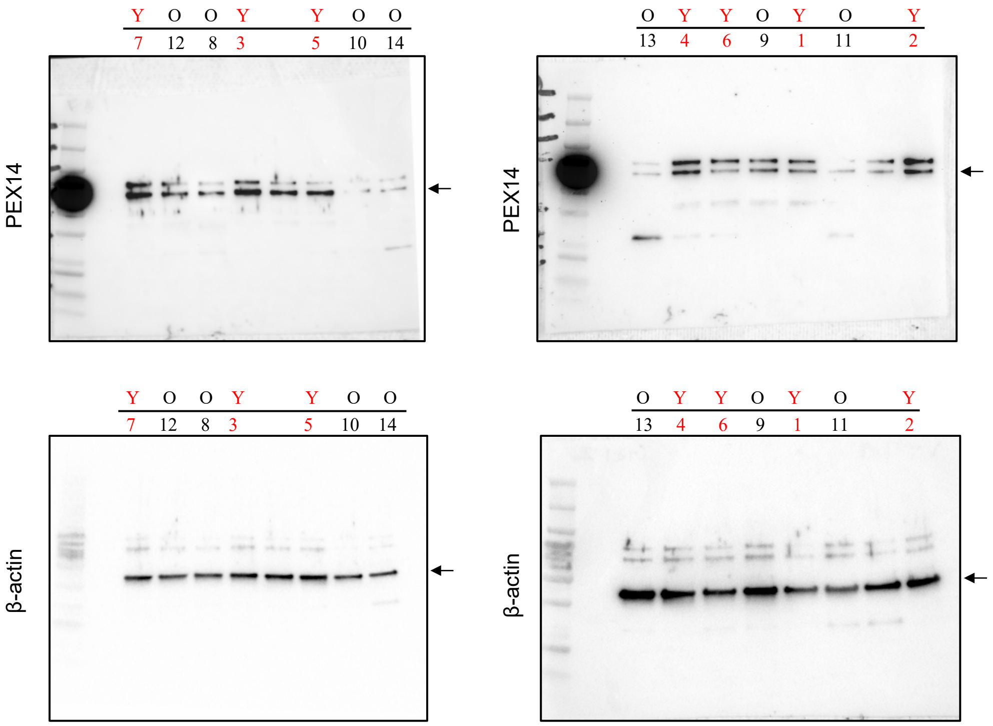

Supplementary Figure 1. Uncropped immunoblots of PEX14 and β-actin in CD19^{+} B cells isolated from young (Y) and old (O) individuals. The number above each immunoblot corresponds to the participant ID of each human subject. Arrows indicate the protein band matching the predicted molecular weight of the target protein.

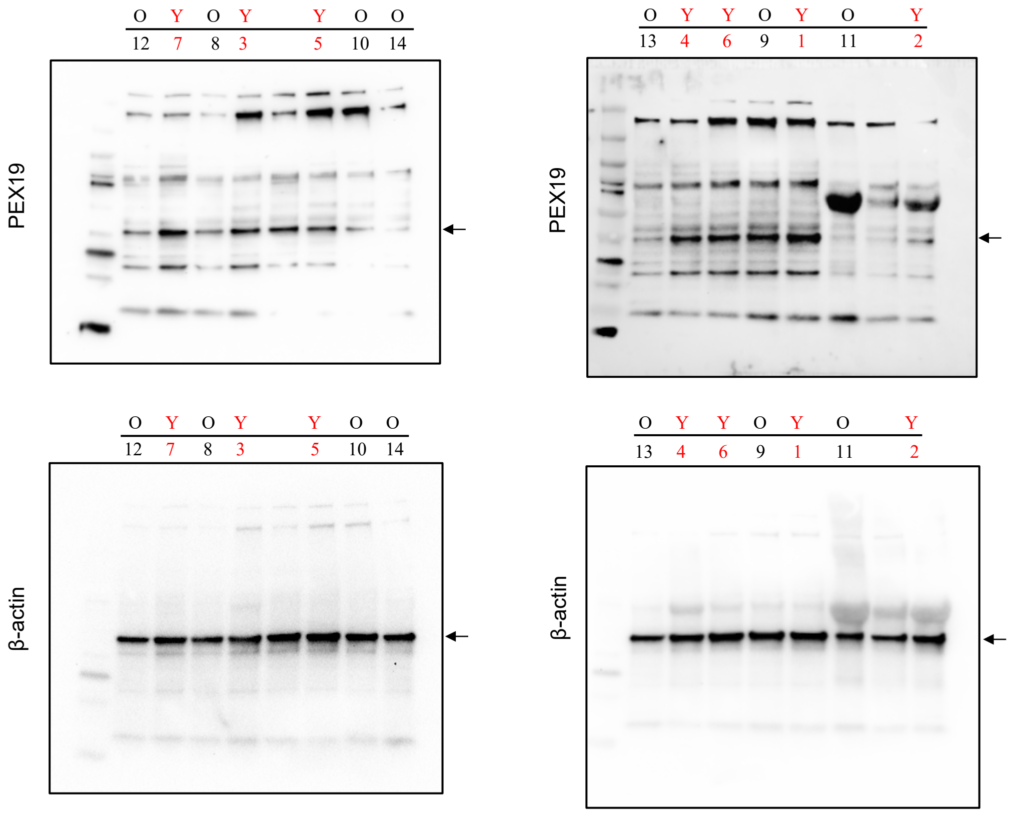

Supplementary Figure 2. Uncropped immunoblots of PEX19 and β-actin in $CD19^+$ B cells isolated from young (Y) and old (O) individuals. The number above each immunoblot corresponds to the participant ID of each human subject. Arrows indicate the protein band matching the predicted molecular weight of the target protein.

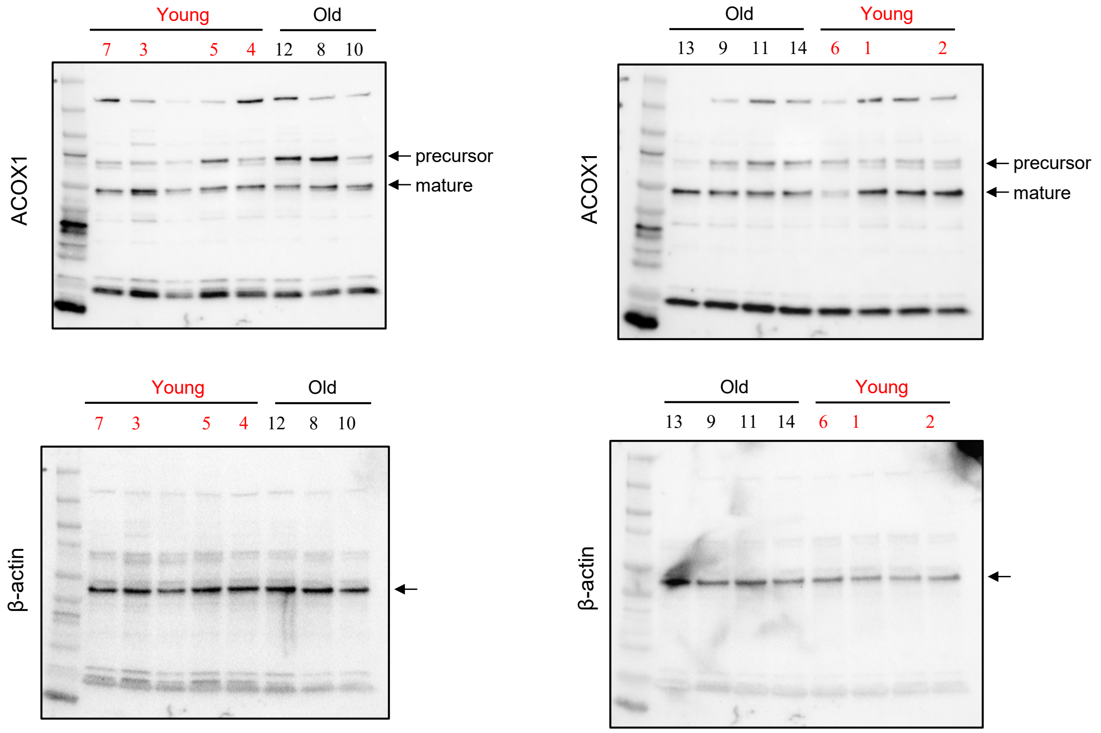

Supplementary Figure 3. Uncropped immunoblots of ACOX1 and β-actin in $CD19^+$ B cells isolated from young and old individuals. The number above each immunoblot corresponds to the participant ID of each human subject. Arrows indicate the protein band matching the predicted molecular weight of the target protein.

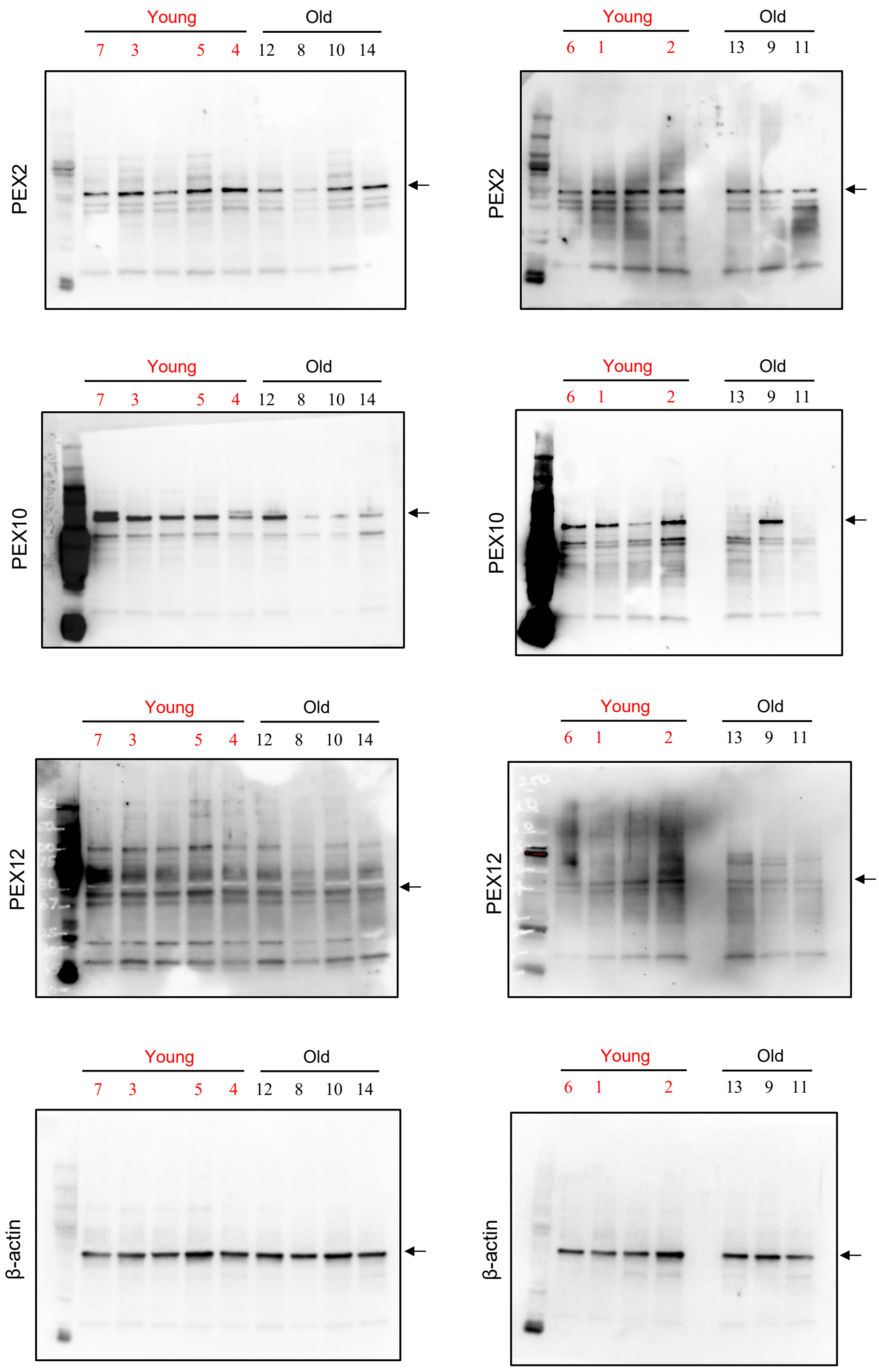

Supplementary Figure 4. Uncropped immunoblots of PEX2, PEX10, PEX12, and β-actin in $CD19^{+}$ B cells isolated from young and old individuals. The number above each immunoblot corresponds to the participant ID of each human subject. Arrows indicate the protein band matching the predicted molecular weight of the target protein.
